# Capacitive Sensors for Label-Free Detection in High-Ionic-Strength Bodily Fluids: A Review

**DOI:** 10.3390/bios15080491

**Published:** 2025-07-30

**Authors:** Seerat Sekhon, Richard Bayford, Andreas Demosthenous

**Affiliations:** 1 Department of Electronic & Electrical Engineering, University College London, London WC1E 6BT, UK; seerat.sekhon.23@ucl.ac.uk; 2 Department of Natural Sciences, Middlesex University, London NW4 4BT, UK; r.bayford@mdx.ac.uk

**Keywords:** electrochemical impedance spectroscopy (EIS), capacitive biosensing, label-free detection, debye length limitation, surface functionalization

## Abstract

Capacitive sensors are platforms that enable label-free, real-time detection at low non-perturbing voltages. These sensors do not rely on Faradaic processes, thereby eliminating the need for redox-active species and simplifying system integration for point-of-care diagnostics. However, their sensitivity in high-ionic-strength solutions, such as bodily fluids, is limited due to a reduced Debye length and non-specific interactions. The present review highlights advances in material integration, surface modification, and signal enhancement techniques to mitigate the challenges of deploying capacitive sensors in biofluids (sweat, saliva, blood, serum). This work further expands on the promise of such sensors for advancing liquid biopsies and highlights key technical challenges in translating capacitive systems to clinics.

## 1. Introduction

The early and accurate detection of disease biomarkers in body fluids is critical for point-of-care diagnostics and personalized medicine. Biosensors, comprising biorecognition elements, transducers, and signal processors, enable this by translating biomolecular interactions into measurable signals. Among these, label-free electrochemical sensors have gained traction due to their low cost, rapid response, and minimal sample preparation. Unlike label-based approaches that require chemical tags, label-free systems simplify workflows and preserve native biomolecular interactions [1].

Electrochemical impedance spectroscopy (EIS) is a non-destructive, label-free technique that probes interfacial phenomena between electrodes and analytes. EIS offers two primary transduction modes for biosensing based on Faradaic (charge transfer resistance ($R_{ct}$)) and non-Faradaic (capacitance ($C_{dl}$)) transduction. Each mode is governed by distinct interfacial dynamics [2]. $R_{ct}$-based sensing relies on the interaction of redox probes, typically ferricyanide or similar species, dispersed in the solution. These probes undergo electron transfer at the electrode interface. When target biomolecules bind to the functionalized electrode surface, they can sterically block or hinder the electron exchange, resulting in an increased $R_{ct}$. This method is well suited for large molecular targets such as proteins or whole cells, where the binding event significantly obstructs electron flow. However, $R_{ct}$ measurements can be susceptible to noise from non-specific adsorption and environmental drift, especially in complex fluids like serum or saliva. Moreover, for low-molecular-weight analytes with a small target-to-receptor size ratio, the physical changes at the interface may not be sufficient to produce a measurable shift in $R_{ct}$, limiting the method’s sensitivity.

Capacitive sensors monitor changes in double-layer capacitance without relying on redox probes or DC bias and are therefore ideal for sensitive, reagent-free diagnostics [2]. The choice between ($R_{ct}$) and ($C_{dl}$) as the primary sensing parameter depends on several factors. A critical consideration is the size of the target biomarker. Larger analytes that cause substantial steric hindrance at the electrode interface are typically more suitable for $R_{ct}$-based detection. Furthermore, electrode design, SAM composition, and immobilization strategies can shift performance in favor of one mode over the other.

Capacitance methods, however, offer greater flexibility for tuning sensitivity through interface design. Though their performance is significantly challenged in high-ionic-strength environments such as blood, serum, or saliva. These media reduce the Debye length to a few nanometers, limiting effective signal transduction from target–receptor interactions occurring beyond the electrical double layer. Additionally, biofouling, non-specific adsorption, and signal drift due to environmental and interface instability contribute to high noise and reduced reproducibility.

To address these limitations, researchers have pursued various strategies like advanced electrode designs (e.g., interdigitated, 3D, nanoporous); novel surface chemistries for antifouling and stable bioreceptor immobilization; interface engineering to control charge density, hydrophobicity, and layer conformation; use of alternative electrode materials like boron-doped diamond (BDD); and conductive polymers to enhance stability and reduce background interference [1]. Moreover, recent integration of EIS with wearable platforms and microfluidic systems opens new avenues for continuous, in situ monitoring of physiological markers. Capacitive sensing, being inherently reagent-less and scalable, holds unique advantages for real-time, non-invasive monitoring applications [3].

This review presents a comprehensive analysis of capacitive biosensors utilizing electrochemical impedance spectroscopy (EIS) for label-free detection in complex biological fluids. It explores advancements in transducer architecture, material engineering, surface functionalization, and signal amplification strategies. Particular emphasis is placed on the operational challenges posed by high-ionic-strength environments and the innovative approaches developed to address them. To the best of our knowledge, this is the first review focused exclusively on capacitive EIS biosensors within such physiologically relevant media.

## 2. Basic Principles of Capacitive Detection

**Figure 1:**
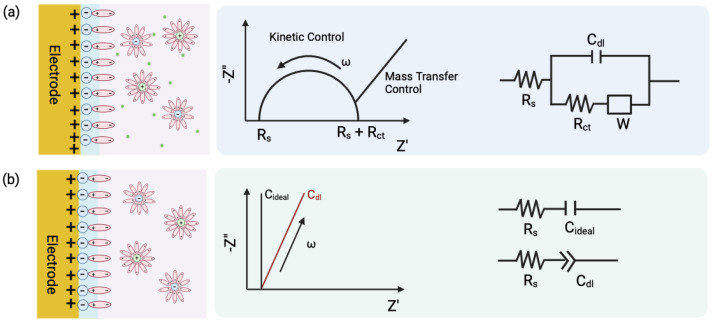
(**a**) Faradaic and (**b**) Non-Faradaic processes at the electrode-electrolyte interface and their equivalent electric circuit representation. Randles circuit is representative of Faradaic processes, where $R_{s}$ is the resistance to redox species in the electrolyte, $R_{ct}$ is the resistance due to charge transfer between the redox species and the electrode surface, and $C_{dl}$ is the capacitance due to the double layer formed at the interface.

**Figure 2:**
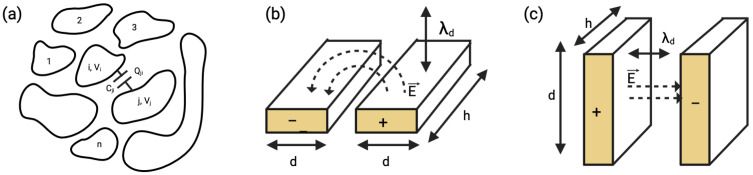
(**a**) Fundamental representation of capacitance between conductors, (**b**) capacitive sensor structure and electric field distribution in 1D coplanar interdigitated configuration (IDC), (**c**) 2D parallel plate capacitor, where $\lambda_{D}$ represents the Debye length.

‘Capacitance’, a term originally coined by Maxwell in 1873, is defined as the quotient of charge induced on one electrode ($Q_{ji}$) due to the potential difference induced by the other ($\left(V_{j}-V_{i}\right)$) (Figure 1a) [4]. The fundamental concept of capacitance and field distributions in interdigitated and parallel-plate configurations is illustrated in Figure 2. Capacitance between electrodes ($C_{ij}$), *i* and *j*, can therefore be formulated as

(1) $$C_{ji}=\frac{Q_{ji}}{V_{j}-V_{i}}$$

For diagnoses in bodily fluids, capacitive sensing is fundamentally based on the principle of charge accumulation on electrodes separated by a dielectric medium of fluids, such as blood, plasma, or urine. These high-ionic-strength fluids limit the effective region within which an electric field can recognize analyte–sensor interactions (termed Debye length screening, defined later in the section). Therefore, it is essential to design sensing topologies that ensure analyte binding occurs within this localized region from the electrode surface. In 1986, Newman et al. were the first to report an affinity sensor based on a capacitive transducer [5]. They demonstrated that the sensing principle relied on changes in dielectric properties, charge distribution, dimension, and shape when an antibody–antigen complex formed on the surface of an electrode. This early work established that capacitive detection is influenced by the design of the transducer, particularly the geometry and distance of electrodes, and the permittivity of the medium.

These parameters can vary the uniform and fringing electric fields of a transducer, which can be critical to the sensor’s performance. A uniform-field capacitance ($C_{u}$) forms when two charged plates face each other in a parallel plate capacitor. For square plates with length (*l*), separated by a distance (*d*), this can be given by the Equation (2), where $\varepsilon_{0}$ is the permittivity of free space, $\varepsilon_{r}$ is the relative permittivity (dielectric constant) of the medium, *A* is the area of the electrodes, and *d* is the distance between the electrodes. This formula is crucial for understanding how changes in the dielectric medium affect capacitance.

(2) $$C_{u}=\frac{\varepsilon_{0}\varepsilon_{r}A}{d}=\frac{\varepsilon_{0}\varepsilon_{r}l^{2}}{d}$$

In contrast, fringing capacitance ($C_{f}$), which is the capacitance that occurs at the edges of the electrodes, is particularly advantageous in biosensing. The fringing field penetrates the immediate vicinity of the electrode surface and interacts strongly with surface-bound molecules or ions in solution [6]. This allows for the detection of changes at distances ranging from nm to μm. Such localization is crucial for monitoring biomolecular interactions like antigen–antibody binding or nucleic acid hybridization. Such fringing capacitance can be given by Equation (3), where $k_{1}$ and $k_{2}$ are empirical constants that depend on the specific geometry and materials of the system. The term (*d*) represents the distance between electrodes, while $C_{p}$ is the parallel plate capacitance per unit area between the electrodes. The characteristic length *l* refers to the dimension related to the length of the electrode or the region where fringing effects are significant. *R* is the effective radius or characteristic length used to approximate the shape of the electrode, and π is the mathematical constant appearing in the logarithmic term.

(3) $$C_{f}\approx k_{1}dC_{p}\ln\left(\frac{k_{2}\pi R}{l}\right);\;R=\sqrt{\frac{l^{2}}{\pi }}$$

The dielectric layer is a critical component of EIS sensors, as it directly affects the capacitance and sensing ability. For instance, the use of SiO_2_ as a dielectric material has been widely explored due to its high stability and compatibility with silicon substrates. Research has shown that texturing the SiO_2_ surface with silica particles of varying sizes (70 nm, 135 nm, and 475 nm) can significantly enhance pH sensitivity. The smallest particles (70 nm) achieved a sensitivity of 52.4 mV/pH, outperforming the planar surface, which only achieved 37.1 mV/pH [7]. This improvement is attributed to the increased surface area and surface defects, which enhance ion adsorption. Similarly, the incorporation of cerium (Ce) into TiO_2_-based dielectrics has been shown to enhance pH sensitivity. A Ce concentration of 5% in CeYTixOy films resulted in a Super-Nernstian response of 66.59 mV/pH, with excellent linearity and reduced hysteresis [8]. This highlights the potential of doping dielectric materials to improve their electrochemical properties.

The capacitive sensor topology is further dictated by the medium of detection, senor-to-target distance, dielectric constant of the target and the applied frequency. Most equivalent circuit models become redundant to account for the sensor behavior of systems measuring capacitance at voltages above the thermal potential (∼25 mV at 20 °C) or at high frequencies [4]. At low frequencies, measurements primarily focus on changes in the double layer’s charge, which is ideal for large surface area electrodes. However, this comes at the cost of lower detection limits, as signals are averaged over a broader range. Additionally, by using current pulses instead of potential pulses, the sensor system reduces the likelihood of damaging the affinity layer, thus preserving the sensor’s integrity and ensuring more accurate and reliable measurements. Based on these parameters, state-of-the-art capacitive sensors can therefore be broadly classified as electrodes based on potentiostatic measurements, interdigitated electrodes (IDEs), and vertically paired electrodes (VPEs).

### 2.1. Electrodes Based on Potentiostatic Measurements

**Figure 3:**
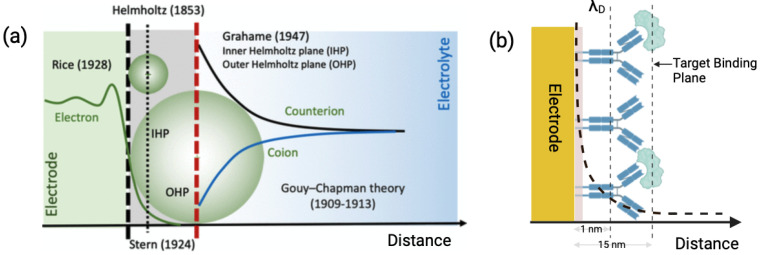
(**a**) Classical theories and models representing EDL formation at the electrolyte–electrode interface [9]. (**b**) Schematic representing debye length limitation with antibody-modified electrodes, where the size of the antibody (15 nm) is larger than the calculated debye length in high-ionic fluids (<1 nm).

The signal transduction in such capacitive sensors is based on the theory of electrical double layer (EDL). When an electrode is immersed in an electrolyte solution, the charged species align at the electrode–solution interface, forming an EDL. The electrode behaves like a capacitor by storing charge ($q_{m}$), such that the solution holds an equal and opposite charge ($q_{s}$), where $q_{m}=-q_{s}$. Closest to the electrode surface, solvent molecules and specifically adsorbed species make up the compact Helmholtz plane, or Stern layer. Solvated ions can only approach the electrode surface at a distance of a monolayer of these oriented solvent molecules (Figure 3a).

**Figure 4:**
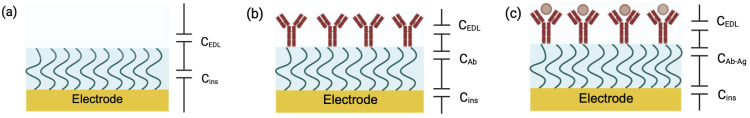
Schematic representation of a model of three capacitors in series that define the total capacitance for an electrode–solution interface biosensor. (**a**) $C_{ins}$ corresponds to the capacitance of the insulating layer and linker molecules attached for directed antibody immobilization, while $C_{EDL}$ represents the electrical double layer, (**b**) $C_{Ab}$ involves the contribution of the capture probe, and (**c**) $C_{Ab-Ag}$ considers the interaction with the antibody and the analyte.

For constructing a capacitive sensor, the electrode surface is usually covered with an insulating layer (a SAM or polymeric layer), and the capture probe is then immobilized. The introduction of capture probes on the electrode surface will modify the thickness and total charge of the EDL, along with the effective permittivity of the solution. These properties are further modified on probe–target binding. Most capacitive sensors discussed in the literature follow this approach. This configuration pushes solvated ions and water molecules farther away from the electrode surface, causing a change in capacitance. $C_{tot}$ is primarily influenced by the smallest capacitance value among the components and can be given by Equation (4). Therefore, a thin insulating layer with high dielectric constant is preferred for changes due to $C_{Ab-Ag}$ to dominate. The component contributions to $C_{tot}$, including $C_{ins}$, $C_{EDL}$, and biomolecular binding layers, are illustrated schematically in Figure 4, which shows the multilayer capacitance model at the electrode–electrolyte interface.

(4) $$\frac{1}{C_{tot}}=\frac{1}{C_{ins}}+\frac{1}{C_{Ab-Ag}}+\frac{1}{C_{EDL}}$$

Gold electrodes have traditionally served as the standard in electrochemical biosensors, but limitations such as susceptibility to biofouling and limited long-term stability in biological media have motivated the exploration of alternative materials. Emerging candidates, including boron-doped diamond (BDD), graphene, platinum (Pt), and PEDOT:PSS, offer improvements in electrical conductivity, chemical robustness, and resistance to nonspecific adsorption. Their integration into capacitive EIS systems has opened new avenues for high-sensitivity, long-duration biosensing in complex matrices.

Among these materials, graphene stands out for its exceptional conductivity, stemming from its high carrier mobility and surface area. Functionalization with aromatic linkers like 1-aminopyrene further enhances charge transfer, enabling detection of targets at femtomolar concentrations in nanosensors [10,11]. Platinum, when dispersed as nanoparticles onto graphene or BDD surfaces, acts as a catalytic interface that accelerates electron transfer, as seen in CRP and glucose immunosensors [12,13]. BDD, with its tunable boron content, combines a wide potential window and low background noise—ideal for detecting electroactive species in applications such as pesticide monitoring [14]. PEDOT:PSS, a flexible conductive polymer, enhances interfacial conductivity and has shown synergistic effects when combined with graphene in glucose and dopamine sensors [15,16].

Equally important is the resistance to biofouling, particularly in biosensors operating in protein-rich environments. PEDOT:PSS offers antifouling performance through its hydrophilic, negatively charged surface that inhibits nonspecific binding [17,18]. BDD has a chemically inert, sp^3^-bonded carbon structure which naturally resists fouling, without the need for complex modifications [14]. Graphene’s planar hydrophobicity can limit fouling, and functional groups such as pyrene derivatives enhance specificity by preventing unwanted adsorption [1]. Platinum, while susceptible on its own, exhibits improved antifouling when used in graphene-based composites [8].

Long-term stability in biological media is another critical factor. PEDOT:PSS maintains electrochemical integrity over time, especially when blended with carbon nanomaterials [16,19]. Graphene’s chemical inertness and mechanical strength preserve sensing performance under repeated use [11]. BDD electrodes demonstrate unmatched stability in harsh environments, retaining performance in extended biofluid exposure [14]. Platinum, integrated with robust matrices like graphene, supports high-fidelity detection with minimal degradation over multiple cycles [12].

These materials have each found success in specific biosensing applications: graphene in DNA hybridization assays and immunosensors [1]; platinum in CRP and enzymatic glucose detection [13]; BDD in environmental pesticide monitoring [14]; and PEDOT:PSS in bacterial metabolite, glucose, and neurotransmitter sensing [18,19]. Their distinct properties and compatibility with capacitive EIS platforms make them critical to the development of next-generation, label-free biosensors optimized for biofluids.

### 2.2. Interdigitated Electrodes (IDE)

**Figure 5:**
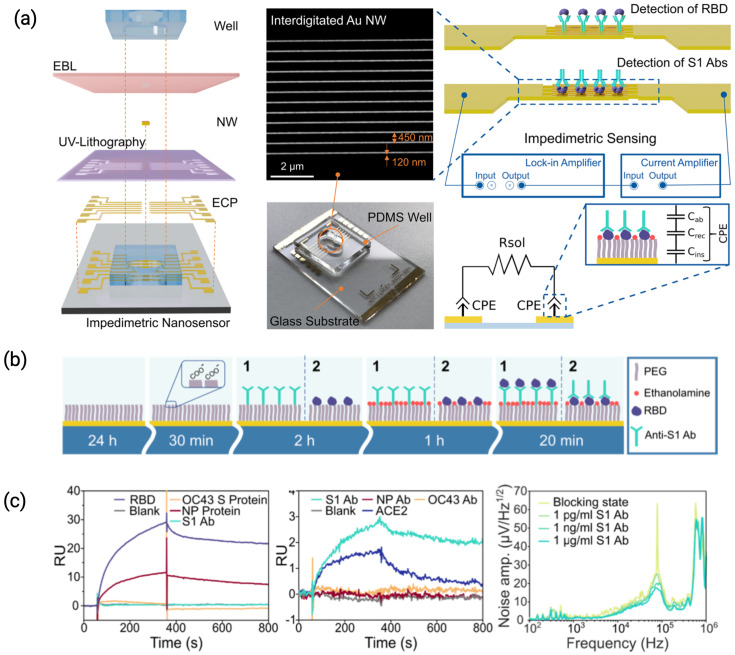
(**a**) Schematic representation of impedimetric nanobiosensor fabrication and assembly, SEM image of interdigitated Au NWs with an average width of 120 nm, length of 49 μm, and spacing of 450 nm, followed by a complete sensor including a PDMS well of 100 μL volume for drop testing. Schematic representation of the measurement setup, with electrical equivalent circuit of the impedance model highlighting constant phase element (CPE) shown on the right. A CPE is a simplified circuit element describing the equivalent contribution of different molecular layers attached on top of the Au NW surface. (**b**) Protocol of surface functionalization for the detection of antibodies, starting with a SAM formation, followed by amine coupling of the captured molecules and surface blocking with ethanolamine. (**c**) The change in the response units (RU) indicates binding of analytes to the sensing surface [28].

In an IDE configuration, the sensor surface constitutes multiple electrode pairs separated by a dielectric surface and provides the intrinsic advantage of a high signal-to-noise ratio [20]. They are usually fabricated using lithography on glass substrates or silicon wafers, with their width and spacing ranging from nm to μm. Such designs generate dense fringing fields by interleaving opposing electrode ‘fingers’. This configuration amplifies the sensitivity to dielectric changes near the electrode surface and the resulting change in capacitance [21,22]. The fabrication and operational features of a nanostructured IDE-based biosensor, including electrode geometry, surface functionalization, and impedance modeling, are illustrated in Figure 5. As capture probes have a lower dielectric constant compared to water molecules, the variation in capacitance on their immobilization is more sensitive than potentostatic measurements.

The first capacitive sensor in an IDE configuration was reported by Newman et al., where two copper conductors (25 mm high and 50 mm wide) were positioned on an insulating material with a 50 mm gap and insulated by a 1 mm layer of parylene polymer [5]. Additionally, a 0.3 mm thick layer of SiO_2_ was vacuum deposited onto the polymer to provide suitable functional groups for the immobilization of the recognition element. Several studies have shown that reducing the finger width and gap can drastically improve sensitivity and detection limits, as this enables a higher concentration of the electric field, improving the signal-to-noise ratio (e.g., IDEs with 50 μm gaps achieve superior sensitivity compared to 100 μm designs [23]). The sensitivity of capacitive detection using IDEs is therefore proportional to the electrode area and number of electrode pairs, and inversely proportional to the electrode gap [24,25,26].

Recent innovations in IDE design have explored non-planar and three-dimensional architectures to address the limitations of conventional planar electrodes. A notable example is the ESSENCE platform, which employs a non-planar, flow-through interdigitated electrode (NP-IDμE) structure packed with functionalized nanomaterials to form a porous capacitive interface [27]. This configuration enhances analyte transport via shear-driven convection, significantly improving sensitivity and reducing response time, particularly in high-ionic-strength media. The porous structure also extends the active sensing volume beyond the classical Debye length limitation, enabling more efficient detection of both small molecules and large biomolecules. Moreover, the ability to tailor the packing material within the microfluidic channel allows for modular adaptation to different targets, making ESSENCE a versatile approach to label-free biosensing.

Nano-structured adaptations of IDEs, such as nanogap capacitors with inter-electrode separations smaller than the Debye length, have been reported to improve alignment with biomolecule dimensions [24]. When modified with thiol-functionalized single-stranded DNA aptamers, the sensor demonstrated high selectivity and sensitivity in detecting human alpha thrombin. Hadi Mirzajani et al. designed a microneedle array integrated with IDEs (MAIDE) as a fully biodegradable platform for wearable and implantable capacitive biosensing applications [29]. This device could track interfacial capacitance variations for different concentrations of bovine serum albumin (BSA) in ISF, demonstrating detection limits as low as 21 ng/mL. The platform’s biodegradable nature addresses long-term implantation concerns, making it a feasible choice for wearable diagnostics and minimally invasive clinical applications.

### 2.3. Vertically Paired Electrodes (VPE)

**Figure 6:**
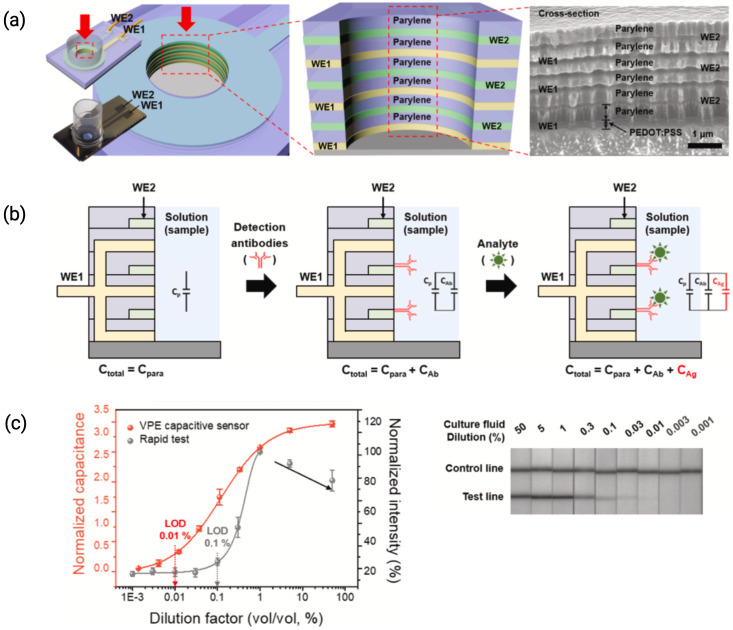
Fabrication of VPEs with three pairs of working electrodes (WE) (**a**) Structure of a VPE and its SEM image (**b**) Analog circuit of vertically paired capacitive sensor for the detection of protein adsorption (**c**) Standard curve for the detection of SARS-CoV-2 in stock diluted with negative stock and comparison of assay results between the capacitive biosensor and the commercial rapid test [26].

A vertically paired electrode (VPE) consists of pairs of stacked electrodes and dielectric layers and has been recently reported to effectively reduce the electrode gap and increase the capacitive biosensor sensitivity [30,31,32,33]. These electrodes are typically made from a conductive polymer, such as PEDOT:PSS, layered with a dielectric material like parylene. The dimensions of VPEs are characterized by reduced electrode, usually gap of less than 1 μm, which enhances sensitivity, and they can consist of multiple electrode pairs, often ranging from two to ten pairs, depending on the design. Figure 6 illustrates the structure of a representative VPE sensor, its analog sensing circuit, and performance in detecting SARS-CoV-2, showcasing the diagnostic potential of this architecture. The total capacitance change ($C_{total}$) caused by the binding of analytes on VPEs can be estimated by Equation (5). $C_{para}$ represents the parasitic capacitance of VPE, $C_{Ab}$ is the capacitance change after immobilization of the antibody layer, and $C_{Ag}$ represents the capacitance change caused by the binding of target analytes [33].

(5) $$C_{total}=C_{para}+C_{Ab}+C_{Ag}$$

Lee et al. were the first to report a capacitive sensor using VPEs that could detect antigen-antibody interactions using anti-HRP antibodies and C-reactive protein (CRP) as model analytes [30]. The Au layers (50 nm and 100 nm) were sputtered onto a glass substrate and were separated by a 550 nm parylene-C dielectric, encapsulated with SU-8, and then exposed via etching. Park et al. demonstrated that VPEs with immobilized anti-SARS-CoV-2 antibodies could effectively detect the virus in viral culture fluids with a limit of detection (LOD) as low as 0.01% [26]. The electrodes reported were made using a PEDOT:PSS conductive polymer layer instead of Ag and Pt metal electrodes, as it allowed for higher fabrication yield due to its easy removal through reactive dry etching.

## 3. Challenges in Deploying Capacitive Sensors in Biofluids

A sensor’s performance is usually evaluated by a standardized criteria which then determines its applicability. Key parameters include resolution, which is the smallest detectable change in target concentration, sensitivity, selectivity, limit of detection (LoD) and sensor noise. Resolution of a sensor is quantified as α/SNR, where α is a confidence parameter, and SNR is the signal-to-noise ratio. Sensitivity refers to the average change in output per unit change in analyte concentration. Selectivity, the ability of the sensor to distinguish the target molecule from others in the sample plays a major role in determining the noise level. Other important metrics include the limit of detection (LoD), the lowest concentration reliably detectable by the sensor, and the dynamic range, which is the ratio of LoD to the highest detectable concentration.

The challenges in deploying capacitive sensors in biofluids emphasize the interdependence of these performance metrics on practical considerations. The Debye length limitation constrains the sensor’s resolution and sensitivity, non-specific interactions affect its selectivity and noise levels, and issues with longevity influence its LoD. Addressing these challenges demands a comprehensive approach to sensor design and therefore asks for advances in material integration, robust surface chemistries, and signal enhancement techniques; discussed in the following sections.

### 3.1. Debye Screening Limitation and Mitigation Strategies

The Debye length ($\lambda_{D}$) is a measure of the characteristic distance over which electric potentials are screened by mobile ionic species in an electrolyte. It is a function of the electrolyte’s ionic strength and can be formulated as Equation (6). In biofluids, which are typically high-ionic-strength environments, the debye length is reduced to just a few nanometers. This compression can obscure the signal from target analytes located beyond the effective sensing zone, thereby limiting resolution and sensitivity. The interplay between sensor geometry, surface functionalization, and debye length must be optimized to maintain reliable detection in such challenging environments.

(6) $$\lambda_{D}=\sqrt{\frac{\varepsilon_{r}\varepsilon_{0}k_{B}T}{2N_{A}e^{2}I}}$$

where *I* is the ionic strength of the electrolyte, $\varepsilon_{r}$ is the dielectric constant, $\varepsilon_{0}$ is the permittivity of free space, $k_{B}$ is the boltzmann constant ($1.38\times 10^{-23}\;J/K$), *T* is the temperature in degrees Kelvin, $N_{A}$ is avogadro’s number $\left(6.022\times 10^{23}\right)$, and *q* is the elementary charge. Debye length is therefore inversely proportional to the ionic strength of the fluid. In blood or serum, typical ionic strengths range from 0.15 to 0.2 M, corresponding to debye lengths of <1 nm. This distance is far smaller than the size of most antibodies or capture probes (2–10 nm), implying that the electrical changes associated with probe–target binding occur outside the electrostatically active region and are being masked. This concept is illustrated in Figure 3, which shows both classical electric double-layer models and the spatial constraints between antibody size and the debye length under physiological conditions.

#### 3.1.1. Electrode Nanostructures

One effective strategy for mitigating Debye screening effects in non-Faradaic and capacitive systems is electrode nanostructuring (nanopores, nanogaps, or nanosurfaces). The geometric confinement and minimal electrode polarization effects inherent to these nanostructures significantly amplify the electric field strength, allowing biomarker-induced perturbations to overcome ionic screening [34]. Moreover, the increased surface area provided by these nanostructures supports a higher density of biomarker binding sites, culminating in a stronger cumulative capacitive signal. These materials also enforce nanoconfinement, ensuring biomolecular interactions occur within the Debye length, thus limiting the masking effects of mobile ions. Table 1 summarizes a range of nanostructured electrode architectures, highlighting their classification, mechanisms of enhancement, and reported sensitivities in complex biofluids.

Significant progress in the development of metal nanogap biosensors has been demonstrated in studies utilizing sub-10 nm electrode separations to probe electrical transport through biomolecules. Notably, Dekker et al. [35] characterized charge conduction through individual 10.4 nm long double-stranded poly(G)-poly(C) DNA strands bridged across platinum nanoelectrodes with an 8 nm gap, offering early insights into the feasibility of direct DNA-based electrical detection. Building on these advances, Hashioka et al. [36,37] employed conventional photolithography to fabricate planar metal nanogap devices with sub-50 nm resolution. Initial nanogap DNA sensors utilizing titanium electrodes exhibited limited signal output (1nA), primarily due to insufficient molecular anchoring. Enhanced performance was later achieved using asymmetric Au–Ti electrode pairs, where thiolated DNA formed robust self-assembled monolayers on gold [38]. This modification led to a tenfold increase in current, highlighting the pivotal role of electrode material and surface chemistry in optimizing nanogap biosensor sensitivity.

**Table 1:** Electrode nanostructures for enhanced sensing in complex biofluids.

Nanostructure	Class	Mechanism	Sensitivity/LoD	Units	Ref.
Au Nanogap Arrays	Plasmonic (0D/2D)	LSPR	1900 nm/RIU; 3.6 nM	RIU, nM	[39,40]
Silicon Nanowires	1D Semiconductor	FET	0.51 ag/mL	ag/mL	[41]
Carbon Nanotubes	1D Conductor	π–π coupling	10 fM	fM	[42]
3D Graphene Foams	3D Hierarchical	Electrochemical	0.5 pM	pM	[43]
Metal Nanogaps (Pt, Au–Ti)	Electrical (0D)	Tunneling	1–10 nA; 1 fM	nA, fM	[35,36,37]
Micro-gap PPE (EIS)	Electrical (2D)	Uniform field	10 fg/mL	fg/mL	[44]
ZnO Nanoporous Electrodes	Semiconductor (3D)	Nanoconfinement	<10 fM	fM	[45,46]
Nanowell EIS Systems	Electrical (3D)	Confined pores	<1 fg/mL	fg/mL	[47]
Vertical Nanogaps	Electrical (3D)	Vertical field	1 fg/mL; 20,000% ΔG	fg/mL, %	[48]
Tubular 3D Nanochannels	Microfluidic (3D)	Biomimetic flow	Enhanced detection	—	[49]

Honda et al. [44] demonstrated the critical role of nanogap electrode architectures in enhancing both the sensitivity and reproducibility of impedimetric biosensors in biofluid environments. By comparing traditional interdigitated electrode (IDE) configurations with a novel micro-gap parallel plate electrode (PPE) structure, they showed that the PPE provided a highly uniform current distribution, minimizing edge-related signal variability. This design enabled the detection of immunoglobulin G (IgG) at concentrations as low as $10^{-14}\;g/\mathrm{mL}$, a record sensitivity for EIS-based IgG detection, while also reducing device-to-device variation.

**Figure 7:**
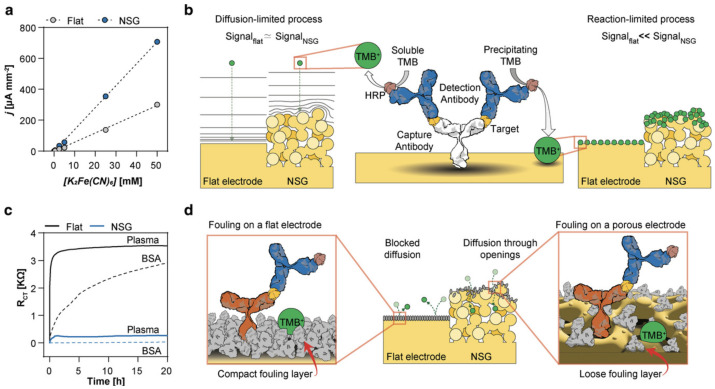
Assay strategy and sample biofouling dependence on the electrode morphology. (**a**) Calibration plot representing peak current densities versus concentration for the electrochemical detection of ferrocyanide on a flat electrode (black circles) versus nanostructured and nanoporous (NSG) electrodes (blue circles) (*n* = 4 independent electrodes). (**b**) Schematic showing two detection strategies based on the use of a diffusible (**left**) or a precipitating (**right**) electroactive compound and the effect of a flat versus a NSG electrode on the signal output. (**c**) Plot of charge-transfer resistance ($R_{ct}$) change over time for a flat electrode (black lines) versus an NSG electrode (blue lines) in 1% BSA (dashed lines) or human plasma (solid lines). (**d**) Schematic showing how biofouling affects the biosensor performance based on the use of flat (**left**) or NSG electrodes (**right**) [50].

Shanmugam et al. demonstrated that nanoporous ZnO electrodes were capable of achieving fM-level sensitivity for protein biomarkers in complex media like serum, owing to the enhanced signal from nanoconfinement effects [45,46]. Selvam et al. further employed nanowell-based EIS systems that utilized spatially confined nanopores to detect NT-proBNP at fg/mL concentrations, further showing the efficacy of this approach in reducing screening contributions and boosting sensitivity [47]. These findings highlight the significance of precise electrode geometry in achieving stable and ultra-sensitive biosensing performance under physiologically relevant conditions. Figure 7 illustrates how nanostructured electrode surfaces enhance signal output and resist biofouling compared to flat electrodes, both in calibration performance and long-term impedance stability in complex media [50].

Kim et al. further optimized vertical nanogap structures (∼20 nm) to achieve fg/mL sensitivity for protein detection [48]. Furthermore, Gao et al. reported a nano-metal–insulator–metal structure capable of detecting DNA hybridization with more than a 20,000% increase in conductance at concentrations as low as 1 fM. These case studies underscore the critical importance of nanogap geometry, electrode composition, and vertical architecture in mitigating Debye screening effects and amplifying bioelectrical signals in complex biofluids.

Three-dimensional (3D) self-assembled tubular nanostructures present promising platforms for replicating vascular-like microenvironments in biosensing. Constructed from materials such as inorganic nanomembranes (${\mathrm{SiO}}_{2},{\mathrm{TiO}}_{2}$), polymers (e.g., SU-8, polyimide, PDMS), and metallic bilayers (e.g., Cr/Au, Ni/Ti), these architectures enhance microfluidic behavior by improving mixing efficiency and enabling size- or deformability-based particle separation [49]. Their biomimetic geometry supports physiologically relevant flow and shear conditions, crucial for accurate analyte detection in complex biofluids. However, a key limitation remains the targeted functionalization of the inner luminal surface, which is essential for specific biochemical sensing. Overcoming this challenge will require innovative surface engineering approaches to fully leverage the diagnostic potential of these 3D bioinspired systems [49].

#### 3.1.2. Reducing Ionic Strength of the Sample

Reducing ionic strength, whether through simple buffer dilutions, layered buffer systems, or advanced electric field techniques, is a widely adopted technique for mitigating Debye screening effects. By lowering the ionic strength (*I*) of the sample, ($\lambda_{D}$) increases, extending the electrostatically active region around the sensor [51]. This enhances the sensitivity of capacitive sensors to biomarker binding events outside the immediate vicinity of the electrode–electrolyte interface.

Daniels and Pourmand (2007) [52] showed that diluting biofluids such as serum or blood at dilution ratios ranging from 1:10 to 1:100 effectively extends the measurable range of impedance changes. This dilution minimizes ionic crowding near the electrode and prevents the shielding of biomarker-induced capacitive changes. Luo et al. (2013) introduced a dual-layer system where a low-ionic-strength buffer was layered directly onto the electrode, isolating the electrode’s EDL from the high-ionic-strength medium, while maintaining the activity of target molecules and allowing ultrasensitive detection of insulin in undiluted serum via non-Faradaic EIS [53]. However, excessive sample dilution can reduce biomarker concentrations to levels below the sensor’s detection limit, requiring careful balancing to maintain both sensitivity and reliability.

#### 3.1.3. Electric Field Modulation at the Interface

Techniques modifying electric fields at electrode–electrolyte interfaces have been employed to extend the effective interaction zone beyond the Debye screening length. By applying a time-varying electric field ($E\left(t\right)=E_{0}\sin\left(\omega t\right)$; $E_{0}$ is the field amplitude and ω the angular frequency), dynamic polarization occurs at the interface such that low-frequency components of the field penetrate deeper into the solution. This field modulation surpasses the usual Debye length constraints, while preserving binding-induced perturbations in interfacial capacitance. Models like the Helmholtz–Smoluchowski and Debye–Hückel formalisms describe charge distribution around an electrode under an electric field [54]. These models emphasize the importance of optimizing the modulation frequency (ω), as it must be carefully selected to account for the ionic composition and dielectric properties of the medium. An ideal modulation frequency should be able to facilitate signal penetration beyond the Debye length, with minimal loss of capacitance sensitivity induced by target binding. The balance between these two factors underpins the utility of electric field modulation in label-free non-Faradaic electrochemical sensors [53].

**Figure 8:**
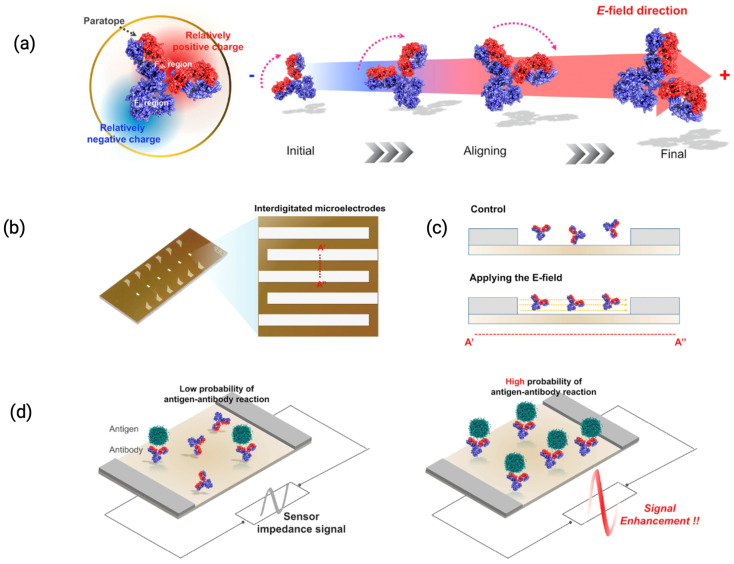
Aligning the orientation of antibodies via modulating electric field. (**a**) The opposite charges of the Fab and Fc regions of IgG influence the antibody’s alignment with the applied electric field. (**b**) A biosensor with interdigitated microelectrodes (IMEs), (**c**) IgG immobilization process to align its orientation. (**d**) Uniformly immobilized IgG on the sensor surface enhances the biosensing signal [55].

Lin et al. (2022) manipulated the electric field across IDEs, which extended the effective interaction range between the sensor surface and the analyte [22]. Figure 8 illustrates how electric field modulation can align antibody orientation via the distinct charges on Fab and Fc regions of IgG. This electric field modulation alters the distribution of ions in the solution, effectively increasing the region over which charge perturbations caused by DNA hybridization can influence the sensor’s interfacial capacitance. They used IDEs operating at high frequencies (up to 90 MHz) to modulate the electric field. At these frequencies, the oscillating electric field perturbs the ionic distribution in the electrode vicinity, thereby diminishing the influence of ions that would otherwise shield the electrostatic interactions near the sensor. The study reported sensitivities of 2.6 fF/log[DNA] and 10.8 fF/log[DNA] at DNA concentrations up to 100 fM in human serum.

EIS-based sensors typically require extended reaction times (often several hours) to ensure that bound molecules reach detectable levels in the absence of enrichment strategies [4,5]. This limitation poses a significant challenge to the adoption of biosensors for on-site diagnostic applications. Lin et al. (2022) applied a 300 mV RMS AC signal over interdigitated microelectrodes (IDMEs) for capacitive sensing [55]. This excitation signal is higher than the typical 5–10 mV RMS commonly used in EIS. The rationale for this modification was to generate sufficiently strong alternating current electrokinetic (ACEK) forces, particularly dielectrophoretic (DEP) forces, to accelerate the transport of analyte towards the IDME surface. The ACEK enrichment effect significantly reduces the time required for testing. As a result, test results were available within 30 s of deployment in serum, demonstrating a substantial improvement in time-to-result for biosensor diagnostics.

### 3.2. Non-Specific Interactions and Sensitivity

Non-specific interactions in the context of biosensing refer to the undesired binding or adhesion of non-target molecules to the sensor surface. These interactions often occur due to weak van der Waal forces, hydrogen bonding, ionic interactions, hydrophobic forces, or electrostatic attraction between non-target entities and the functionalized surface of the sensor. Typical non-specific species include abundant proteins (e.g., albumin in serum), lipids, salts, or other molecules present in bodily fluids but not intended to be detected by the sensor. In capacitive sensors, such interactions alter the electrical characteristics at the electrode-electrolyte interface, inducing changes in the dielectric constant, double-layer capacitance, or overall impedance. A detailed comparison of specific versus non-specific binding characteristics is presented in Table 2, highlighting the role of intermolecular forces and interface architecture in determining binding behavior [56].

The interfacial characteristics of capacitive EIS sensors, particularly surface energy and hydrophobicity, play a critical role in determining their sensing performance and mitigating non-specific interactions in biofluids. These surface properties influence molecular interactions, fouling resistance, dielectric behavior, and ultimately, signal stability and sensitivity.

**Figure 9:**
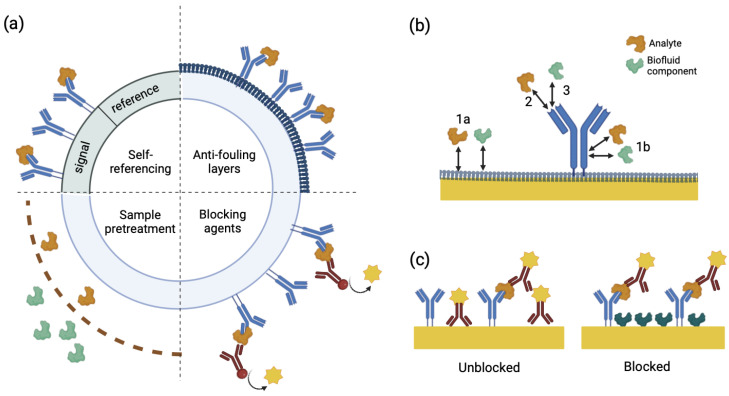
(**a**) Approaches to minimize non-specific binding (NSB). (**b**) Different NSB possibilities on a sensor surface: (1a) direct binding to the surface or (1b) to the capture probe; (3) binding to the paratope of the capture probe by interferents, where (2) represents the specific target–paratope binding. (**c**) Blocking can reduce NSB of the labeled component of a typical immunoassay and primarily reduces type 1a interactions.

Surface energy governs the affinity of the electrode surface toward polar or non-polar species. High-energy, hydrophilic surfaces enhance the adsorption of polar molecules such as water and ions, potentially increasing the local concentration of target analytes and improving sensitivity. Conversely, low-energy, hydrophobic surfaces reduce nonspecific interactions by repelling aqueous components, thereby improving selectivity and minimizing baseline drift. The optimal surface energy depends on the specific application—biomarker capture may benefit from moderate hydrophilicity, while long-term monitoring in protein-rich fluids favors antifouling hydrophobic coatings [57,58]. Figure 9 illustrates common pathways of nonspecific binding and summarizes surface blocking strategies to mitigate such interactions, particularly in immunoassay configurations.

Hydrophobicity, in particular, serves a dual function. It suppresses nonspecific adsorption, a common issue in biological matrices, and also modulates the electrical double layer’s structure, influencing overall capacitance. Hydrophobic self-assembled monolayers (SAMs) have been shown to improve antifouling characteristics in electrochemical aptamer-based sensors, leading to improved stability and lower detection limits [59]. However, excessive hydrophobicity may hinder target interaction or reduce capacitance, especially when the surface repels not only interferents but also the analyte itself [60,61]. Tailoring wettability through controlled surface modification is therefore essential.

From a surface engineering perspective, static and stimuli-responsive coatings represent two distinct classes of interfacial modification strategies aimed at tuning hydrophobicity and surface energy; parameters that critically influence biosensor performance in complex biofluids. Static coatings, including self-assembled monolayers (SAMs), are engineered for physicochemical consistency. Hydrophobic surfaces constructed from alkyl or fluorinated terminal groups, as well as hydrophilic coatings terminating in hydroxyls, have demonstrated long-term robustness under physiological conditions [62]. Ma et al. [63] demonstrated that lipid-inspired fluorocarbon coatings closely mimic the stability of biological membranes and resist delamination in aqueous media. Mousavi and Pitchumani [64] showed that superhydrophobic and lubricant-infused surfaces retain water repellency and corrosion resistance under elevated thermal and hydrodynamic stresses, making them suitable for durable biosensor platforms.

Stimuli-responsive coatings provide dynamic modulation of surface properties through conformational or chemical transitions triggered by external stimuli such as pH shifts (typically in the range of pH 4–9), temperature variations (e.g., across a lower critical solution temperature, LCST, near 32 °C for poly(N-isopropylacrylamide)), or light irradiation (UV–Vis range, 200–700 nm). These adaptive coatings enable reversible transitions between antifouling and bioadhesive states, critical for biosensing applications where switchable analyte capture and release are required. As demonstrated by Otsuka et al., functional polymer brushes and hydrogels tailored with biorecognition elements (e.g., peptides, oligonucleotides) can maintain antifouling performance, with protein adsorption reduced by over 90% relative to unmodified surfaces [65]. However, they also reported that repeated actuation cycles induced measurable surface degradation, especially under exposure to aggressive media such as blood serum or saline solutions with elevated ionic strength (>150 mM NaCl), leading to a decline in switching fidelity and biointerface integrity over time.

To further refine interfacial performance, complementary methods have been adopted. Li et al. [66] demonstrated that oxygen plasma treatments significantly increase surface polarity and wettability in carbon nanotube-based sensors, thereby enhancing bioreceptor immobilization. Bakestani et al. [58] showed that chemical functionalization with carboxylate and hydrophobic moieties enables tunable control over antifouling properties and bioreceptor anchoring.

While static coatings offer long-term structural integrity and consistent antifouling performance, stimuli-responsive systems provide dynamic adaptability that can enhance sensor responsiveness and functionality in fluctuating environments. Ultimately, it is the rational selection and integration of surface modification strategies that dictates the functional efficacy of biosensor interfaces, governing both their analytical sensitivity and operational durability in complex biological environments.

### 3.3. Sensor Regeneration

Goode et al. (2014) defined sensor regeneration as the process of restoring the functionality of a biosensor after it has been used to detect an analyte. This involves overcoming the attractive forces between the bioreceptor and the analyte, allowing the sensor to be reused for subsequent measurements [67]. To facilitate sensor regeneration, the architecture and construction of biosensors must be carefully designed and adjusted in several key ways. Criteria for effective biosensor regeneration are outlined in Table 3, which highlights key performance benchmarks such as signal retention, repeatability, and minimal transducer reconstruction [56].

One crucial aspect is the selection of receptors. Bioreceptors should be chosen based on their stability and ability to withstand regeneration processes. For instance, employing robust enzymes or affinity proteins that can tolerate regeneration conditions without significant degradation is essential. Another important factor is the surface chemistry of the sensor. The surface should be engineered to minimize non-specific binding and enhance the release of analytes during regeneration. This can involve modifying the surface with specific coatings or functional groups that promote the detachment of bound analytes, without damaging the bioreceptor.

Further, the sensor architecture should allow for easy adoption of regeneration buffers that can effectively disrupt interactions between the bioreceptor and the analyte. The design should facilitate the flow of these buffers across the sensor surface, for thorough cleaning and regeneration [54]. Maintaining the mechanical stability of the sensor during regeneration cycles is another crucial parameter. This includes ensuring that the materials used in construction can withstand repeated exposure to various chemical environments without losing their mechanical properties. Incorporating built-in mechanisms for regeneration, such as microfluidic channels for buffer flow or automated cleaning systems, can streamline the regeneration process and make it more efficient.

Saateh et al. (2024) demonstrated a plasmonic oligonucleotide biosensor capable of regeneration within a minute and 100+ reuses per day in undiluted human serum [68]. Similarly, electrochemical-affinity-based sensors integrated into microfluidic organ-on-chip devices have achieved continuous biomarker monitoring for up to 7 days without signal degradation [69,70]. These examples underscore how regeneration-enabled reusability extends biosensor function far beyond traditional single-use designs. Aptamer-based biosensors offer exceptional regeneration capacity due to their thermal and chemical stability. Potyrailo et al. (2015) reported aptamer layers withstanding 365 regeneration cycles with <5% variation in affinity. Their synthetic origin allows precise refolding post-regeneration, making them ideal for wearables, point-of-care platforms, and low-maintenance diagnostics [71].

Gupta et al. (2020) showed that encapsulating antibodies in organosilica matrices preserved activity under detergent-based regeneration such as SDS [72]. Additionally, nanocomposite antifouling coatings have enabled month-long operation in complex fluids such as nasopharyngeal secretions, while maintaining electron transfer kinetics and minimizing fouling [73,74]. These developments enhance both chemical resilience and mechanical robustness, crucial for high-frequency or long-term sensing. Innovations in solid-state and photoactivated regeneration have minimized reliance on external chemicals. For example, electrochemical pH modulation using palladium electrodes enables localized shifts of ±4 pH units, effectively reversing antibody–antigen binding without added reagents [75]. Similarly, photoacid-based systems, such as those using immobilized HPTS, trigger antibody regeneration via light-induced pH changes—an approach validated for heart failure biomarkers like BNP in wearable sensors [76].

### 3.4. Sensor Longevity and Anti-Fouling Layers

Maintaining stability in the presence of biofluid-induced fouling and oxidation processes is essential for a prolonged sensor lifespan. Biofouling, caused by the adhesion of proteins, lipids, or other molecules to the sensor surface, further limits the signal specificity and sensitivity. To mitigate this, antifouling layers are critical for maintaining the stability and functionality of biosensors in complex environments such as blood or serum. Antifouling materials like polyethylene glycol (PEG), zwitterionic polymers, and hydrophilic self-assembled monolayers (SAMs) are frequently employed to create such surfaces that resist nonspecific adsorption, while preserving specific target–receptor binding.

**Figure 10:**
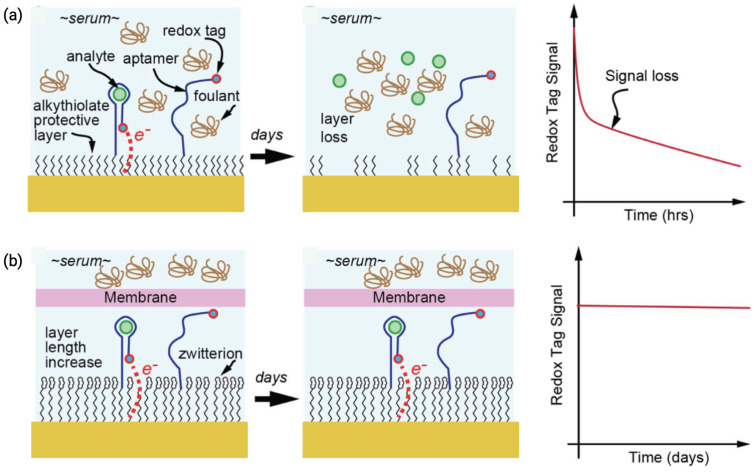
Sensor fouling deployed in biofluids. (**a**) Aptamer sensors usually employ a SAM layer for immobilizing Au electrodes. Here, an alkythiolate-modified aptamer with a terminal redox reporter is shown. In the presence of analyte, the aptamer folds, bringing the redox tag closer to the sensor surface. When deployed in serum, foulants have a tendency to interact with the sensor architecture. Following continuous interrogation, there is extensive monolayer and aptamer loss, leading to loss of the redox tag signal over a timeline of hours. (**b**) The addition of a protective membrane or an antifouling zwitterion monolayer protects sensor interfaces against unwanted fouling. Upon continuous interrogation, the redox tag signal is retained [78].

The first functional antifouling layer adopted in label-free, non-Faradaic EIS biosensors was reported in 2010, where SAMs or silane blocking layers were used to minimize nonspecific binding [47,52]. PEG is among the most widely used antifouling materials, forming a hydrophilic and flexible chain layer on the sensor surface that sterically hinders proteins and other biomolecules. By creating a hydrated barrier, PEG effectively repels fouling agents. Recent advancements have demonstrated that zwitter ionic polymers outperformed PEG, particularly in high-ionic-strength environments like serum [53,77]. Zwitterionic coatings utilize positively and negatively charged groups to attract water molecules, forming a robust solvation layer that resists non-specific adsorption. This dual-charge property enhances stability under a wide range of salt concentrations and pH levels, making zwitter ionic polymers more versatile. Luo et al. demonstrated the effectiveness of such polymers by applying them to gold electrodes, achieving stable antibody binding and robust insulin detection in undiluted serum, while simultaneously resisting fouling over an extended period of use [53]. Figure 10 further illustrates the importance of antifouling membranes or zwitterionic coatings in preserving sensor integrity and redox signal stability during extended exposure to biofluids.

In addition to antifouling coatings, stable covalent attachment methods can also enhance sensor longevity, particularly in challenging biofluid environments. Covalent linking methods like EDC/NHS chemistry ensure strong and durable bonds between bioreceptors (e.g., antibodies or aptamers) and the sensor surface, preventing receptor detachment or degradation during repeated measurements. Liu et al. showcased covalent linkers on gold electrodes, demonstrating consistent sensor responses in high-ionic-strength conditions such as serum, even after multiple cycles of use [79]. This adds significant robustness to biosensors, extending their applicability for clinical diagnostics. Nanostructured sensor platforms (be it nanoporous Au or ZnO) further amplify the benefits of antifouling and stability strategies. Shanmugam et al. combined nanoporous platforms with hydrophilic SAMs or PEG layers, achieving ultrasensitive detection of biomarkers like troponin-T in whole blood, while maintaining stability against fouling [45,47]. This integration of nanostructures with antifouling coatings represents a synergistic approach to improving both signal integrity and sensor longevity.

## 4. Measurement Strategies

The accuracy of EIS-based sensors depends on both sensor architecture and measurement strategies, particularly in mitigating non-specific interactions and environmental noise. A common approach involves using a reference sensor that excludes target binding but mirrors the working sensor’s response to environmental changes. By comparing signals between the two, differential measurement helps suppress external influences such as temperature or pH fluctuations. However, this method relies heavily on the careful selection of the surface functionalization practices, especially in complex sample environments [22,80]. Alternatively, measuring impedance before and after target binding, while controlling external variables, can also isolate signal changes due to specific interactions. This strategy similarly enhances selectivity and sensitivity, particularly when both sensors respond similarly to background interference.

Traditional amplitude-based EIS suffers from noise interference, significantly compromising sensor performance. To overcome this, Chen et al. (2023) introduced an impedance conversion stage that stabilizes signal fluctuations, enhancing repeatability and sensitivity. Building on this, a voltage-to-frequency-to-voltage (V/F/V) conversion method was developed to improve linearity by translating output voltage into frequency shifts, effectively reducing noise and lowering the relative error from 2.26% to 0.0045% thereby enabling highly precise impedance measurements in complex biological fluids [81].

Further improvements include the integration of a liquid-free reference (LR) in differential EISCAP designs, which achieves up to 80% background signal suppression and improves accuracy and long-term pH sensing stability [82]. Additionally, low-frequency EIS has been tailored for graphene-based biosensors, enhancing detection limits and enabling the design of sensor layouts with optimized low-frequency sensitivity [83].

## 5. Advances in Surface Functionalization

The performance of label-free sensors is largely dependent on the affinity of the capture probe and the immobilization strategy adopted. The structure and activity of the probe should be preserved during electrode modification and is a key consideration during construction of capacitive sensors. In biosensing, particularly under widely varying real-time conditions, the reliability and reproducibility of an operation is primarily dictated by the success in fabricating a compact, pinhole-free immobilization layer. Common strategies for immobilization and insulation include self-assembled monolayers (SAMs) of alkyl thiols, polymers, and silanes. However, the conundrum is that ultrahigh molecular-level sensitivity has always been associated with unreliable and irreproducible responses from the sensor and irregularities in the immobilization layer [84].

Since a comprehensive review of biosensor surface chemistry is beyond the scope of this discussion, certain key findings highlighting advances in immobilization strategies, in the context of improved reproducibility for capacitive sensor, are provided in the following sections.

### 5.1. Self-Assembled Monolayers (SAM)

The most prevalent surface modification chemistries in biosensing are based on thiols bound to gold surfaces or siloxanes to oxide surfaces. Most capacitive sensors reported in the literature utilize thiolated SAMs to attach the capture probes to gold electrodes. However parameters such as SAM density, thickness, and terminal functional groups impact their ability to suppress undesired Faradaic processes and maintain a purely capacitive sensing interface.

SAMs with longer carbon chains form more dense monolayers due to hydrophobic interactions of the chains. Usually a SAM with $C_{11}$ or greater gives packed films [52]. For nf-EIS sensors, a tightly-packed (high $R_{leak}$) SAM is desirable, in contrast with f-EIS sensors, where the electrode surface needs to be more accessible to the redox species. Namhil et al. (2019) utilized an SAM as an insulation layer in sensors with nano-gap capacitors. A precisely tuned SAM layer improved signal detection by optimizing surface chemistry and maintaining compatibility with nf-EIS in ionic environments [85]. Yusof et al. (2011) showed that SAMs could stabilize impedance measurements in saline solutions, enabling accurate detection of DNA hybridization events by suppressing polarization artifacts [86].

**Figure 11:**
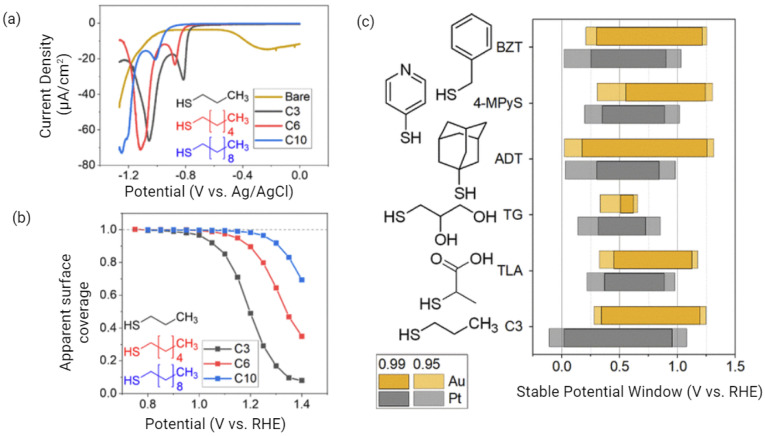
Electrochemical stability of self-assembled monolayers (SAMs) on Au and Pt electrodes. (**a**) Cyclic voltammograms showing reductive desorption behavior of alkylthiol SAMs with varying chain lengths (C3, C6, C10), indicating enhanced stability with longer chains. (**b**) Apparent surface coverage as a function of potential (vs. RHE), highlighting oxidative stability trends; longer chains retain coverage at higher potentials. (**c**) Comparison of stable potential windows for different thiol-based SAMs on Au (gold) and Pt (gray) electrodes, with aromatic and bulky headgroups showing enhanced electrochemical resilience [89].

However, SAM desorption is a major concern for sensor performance and a prime reason why a sensor might have a response to a blank solution. Poorly formed or unstable SAM layers can lead to signal drift, non-uniform coverage, and increased noise. Lai et al. recently provided critical insights into the stability of SAMs during dry storage, concluding that longer-chain SAMs exhibit greater stability [53], which aligns with the electrochemical desorption and oxidation trends observed in Figure 11 for longer-chain SAMs. Furthermore, the use of preservatives was shown to maintain stability for over one month, with reproducible results. However, the stability of SAMs can also be adversely affected by experimental conditions. For example, the hexacyanoferrate (II/III) redox couple, widely used in f-EIS sensors, has been reported to degrade the electrode–SAM interface over time, particularly under light exposure [87,88]. This degradation can also reduce the activity of peptide probe layers [5].

Assaifan et al. (2024) explored SAM functionalization on interdigitated electrodes and reported that intermediate concentrations of cysteamine yielded the best performance, ensuring maximum insulation, while maintaining bioreceptor activity [67]. Boubour et al. reported that over 40 h of incubation was required to form a tightly packed SAM, as determined by observing the purely capacitive behavior at low frequencies [28], but others have reported 15–20 h, depending on SAM composition, and even as little as 2 h [46]. Brothers et al. (2020) demonstrated the influence of SAM chain length and charge density on sensing performance, showing that denser SAMs improve biorecognition by reducing nonspecific interactions and enhancing surface stability [67].

Furthermore, it is important to note that SAMs serve as an effective electrical insulator only within a specific range of DC bias voltages, which is influenced by the chain length and terminal group of the SAM. For instance, gold–thiol SAMs with hydrophilic headgroups exhibit DC conduction (finite $R_{leak}$) even at 0 *V* bias versus Ag/AgCl when employing a $C_{16}$ SAM in the absence of a redox species [79]. This conduction is likely attributed to voltage-induced structural rearrangements in the SAM, resulting in pinholes [85], or to ion and water molecule permeation through the SAM layer [55]. At extreme DC bias voltages, the bonds between the metal electrode and the biological probe layer can undergo oxidation or reduction, with reported values for gold–thiol systems summarized in [52]. Interestingly, this phenomenon has been exploited for selective electrode functionalization [29,77,90,91].

### 5.2. Polymeric Films

Polymer films play a critical role in enabling probe immobilization, either through covalent bonding or physical entrapment. Covalent attachment is facilitated by incorporating functional groups such as carboxyl (-COOH) or amine (-NH_2_) into the polymer, allowing the direct linkage of biomolecules via well-known chemistries such as amide bond formation or cross-linking. For instance Berezhetska et al. demonstrated that PEDOT:PSS functionalized with carboxymethylated dextran can covalently graft capture probes without additional post-treatment steps, providing highly stable biointerfaces [91]. The use of molecular imprinting in polymer films (MIPs) has emerged as a promising technique [92], where target analytes or their mimics are used as templates during polymerization to create highly specific binding sites. After template removal, the polymer retains molecular ‘imprints’, allowing selective rebinding of the target analyte, often enhancing sensitivity and specificity in biosensing applications. An alternative strategy involves the direct entrapment of capture probes during polymerization. Zuo et al. demonstrated the use of Polypyrrole (PPy) to entrap probes directly during electropolymerization [88]. This entrapment ensures stability and allows for signal transduction upon probe–analyte binding events. Kondyurin et al. further developed PPy as a modification layer through plasma immersion ion implantation, enabling direct covalent protein immobilization, which improved sensor reusability, while maintaining a high capacitive response [92].

Most of the polymer films utilized in biosensing are either semiconducting or redox-active, implying they not only act as structural platforms, but also participate in the electrochemical processes at the sensing interface. For instance, conducting polymers such as polypyrrole, polyaniline, and PEDOT exhibit controllable electrical conductivity, making them suitable to be integrated into electrode assemblies as electronic mediators or extensions of the metallic electrode [91,92]. Their semiconducting properties facilitate weak charge transport and, in some cases, enhance the electron transfer efficiency between target species and the electrode surface. PEDOT derivatives, in particular, have gained traction in nf-EIS systems because of their ability to tune conductivity, without compromising the insulation necessary to suppress Faradaic processes [53]. Supramolecular interaction strategies involving PEDOT have allowed the development of functional electrodes for biospecific interactions such as carbohydrate–lectin or enzyme–substrate binding, while maintaining strong signal transduction properties and bioactivity [47].

Polymers containing redox centers, such as conducting polymers doped with catalytic or electron-carrying species can act as ‘bridges’ between target species and electrodes by facilitating long-range electron transport. Rossignatti et al. demonstrated that incorporating nanoparticles such as CeO_2_ or WO_3_ into these polymers improves their dielectric properties and mechanical stability, offering higher sensitivity and reliability in detecting capacitive changes [54,93]. However, in non-Faradaic systems, the potential for these semiconducting polymers to interfere with purely capacitive measurements must be carefully managed. Crosslinking agents, such as glycidoxypropyltrimethoxysilane (GOPS) or silanes, are commonly added to improve the mechanical strength and adhesion of films like PEDOT:PSS to electrode surfaces, reducing the risk of delamination and prolonging usage under hydrated conditions [91,92]. Encapsulation with additional insulating layers, such as graphene oxide or silica coatings, provides protection against moisture ingress and chemical degradation, while also minimizing leakage currents that compromise capacitive signals [79].

Non-conducting polymeric films provide a highly resistive barrier, while simultaneously acting as insulating layers that suppress Faradaic (redox) processes across the electrode–electrolyte interface. Poly(o-phenylenediamine) (PoPD) and polyelectrolyte multilayers are among the widely studied non-conducting polymers for these applications [94]. Gornall et al. (2010) demonstrated the use of such PoPD films on carbon electrodes, showing that the resulting films effectively suppressed Faradaic processes, as evidenced by cyclic voltammetry [94]. Polyelectrolyte multilayers, on the other hand, are fabricated using the layer-by-layer (LbL) assembly approach, which involves alternating deposits of positively and negatively charged polyelectrolytes. This technique allows precise control of film thickness and surface functionalization, leading to tunable dielectric properties. For instance, polyelectrolyte multilayers with nanometric control of bilayer thicknesses revealed a direct correlation between the number of bilayers and the observed capacitance, which increased linearly with their growth [77].

The performance utility of thin non-conducting polymer films is often hindered by defects, such as pinholes, inhomogeneities, or voids within the film structure. These defects may arise from physical imperfections during deposition (e.g., spin-coating or electropolymerization) or incomplete bilayer formation in LbL assemblies. Such issues introduce pathways for ionic or electronic charge transfer, reducing the insulating capacity of the films and increasing the likelihood of leakage currents in high-sensitivity applications. Optimizing the electropolymerization conditions (e.g., monomer concentration, applied potential) was shown to reduce these defects in PoPD films, resulting in smoother and more uniform coatings [94]. Correspondingly, in polyelectrolyte multilayers, improper adsorption of layers during LbL deposition caused heterogeneity, which compromised the uniform capacitance response expected from these dielectric films [53].

### 5.3. Bioreceptor Immobilization

Bioreceptor immobilization plays a pivotal role in dictating the analytical performance of biosensors, especially in complex biofluids. Among the most impactful strategies are those that control the orientation and density of the immobilized antibodies. Baltierra-Uribe et al. (2019) demonstrated that protein-A-mediated orientation of IgG antibodies on silicon surfaces improved Brucella abortus detection, achieving a threefold increase in signal intensity compared to random immobilization [95]. Similarly, Liao et al. (2021) reported enhanced electrochemiluminescence (ECL) output when antibodies were oriented on Fe_3_O_4_/CdTe nanoparticles via protein G, achieving a LOD of 12 ng/mL for human serum albumin [96]. In general, immobilization methods can be categorized as covalent attachment, physical adsorption, and biorecognition affinity-based methods, each with distinct mechanisms and trade-offs [97].The adapted immobilization processes should strike a balance between ensuring strong probe attachment and preserving its structural integrity and bioactivity. Representative immunosensor platforms employing non-Faradaic EIS and various bioreceptor immobilization strategies are listed in Table 4.

**Figure 12:**
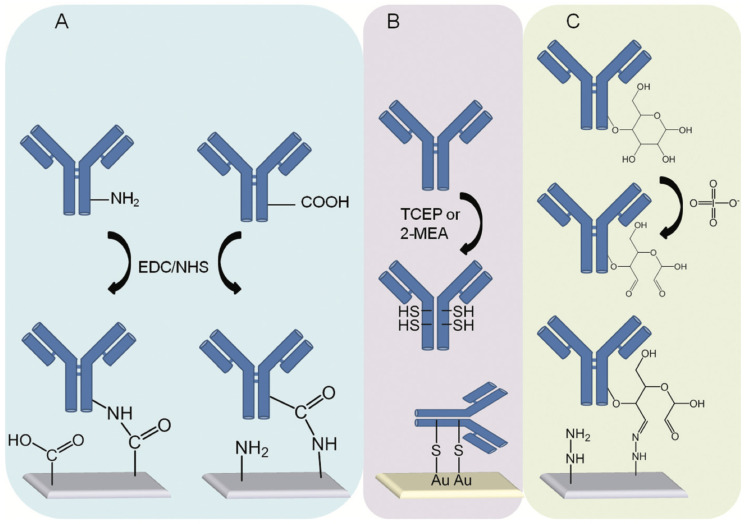
Oriented Antibody Immobilization Strategies. (**A**) EDC/NHS coupling of antibody amine and carboxyl groups to surface carboxyl and amine groups. (**B**) Reduction of antibody disulfides, with TCEP or 2-MEA, to reactive thiols for binding gold substrates. (**C**) Periodate oxidation of carbohydrates in the Fc region of antibodies followed by coupling to hydrazide surface chemistry [97].

Covalent immobilization forms strong, durable bonds between functional groups on the probe and sensor surface. This method prevents probe detachment under harsh conditions, including high ionic strength, fluctuating pH, or elevated temperatures, making it a common strategy in electrochemical sensors [97]. Processes including silanization, glutaraldehyde coupling, carbodiimide chemistry, and click chemistry result in covalent attachment (Figure 12). Silanization involves silane coupling agents like APTES, which functionalize the surface with reactive groups such as amines [125,126]. APTES-modified surfaces can react with aldehyde groups from glutaraldehyde to attach proteins or enzymes via their amine residues. Carbodiimide chemistry, using reagents like EDC and NHS, forms stable amide bonds between carboxyl groups on the sensor surface and primary amines on the bioreceptor, facilitating antibody or enzyme immobilization under mild conditions [125]. Click chemistry, using azide-alkyne cycloaddition, enables highly specific covalent coupling under mild conditions [126], making it particularly suitable for attaching delicate bioreceptors like aptamers or DNA, while preserving their functional integrity.

Physical adsorption relies on non-covalent interactions such as van der Waals forces, hydrophobic interactions, electrostatic attraction, and hydrogen bonding for probe immobilization. It is a straightforward approach and does not require chemical modifications at the sensing surface. As mentioned by Moraes et al., electrostatic adsorption is often used for immobilizing negatively charged probes (e.g., nucleic acids, enzymes) on positively charged surfaces like amino-silanized SiO_2_ or Ta_2_O_5_ layers [127]. By adjusting the pH or ionic strength, surface charge and adsorption strength can be finely tuned. Hydrophobic interactions between nonpolar protein patches and hydrophobic surfaces (e.g., untreated silicon wafers) are another means of adsorption [128]. Physical adsorption’s main drawback, however, is weak binding, making desorption possible under physiological flow or high-ionic-strength conditions. Additionally, nonspecific interactions can increase nonspecific interactions, and with a low signal to noise ratio [127,128].

Bio-recognition-based techniques exploit natural or engineered affinity interactions for bioreceptor immobilization. These interactions are highly specific and reversible, enabling fine control over immobilization and surface regeneration. Protein A or G is commonly used to immobilize antibodies by binding to the Fc region, leaving the antigen-binding Fab region exposed [127]. This method ensures correct orientation and specificity, making it advantageous for immunosensors. Similarly, the biotin–avidin system is widely employed, with biotinylated bioreceptors attaching to avidin-coated surfaces, creating stable, specific bonds. This strategy is adaptable for multiplexed sensors [126]. DNA-based sensors often utilize complementary hybridization, where DNA probes are immobilized through thiol–gold linkages or direct adsorption to functionalized surfaces, allowing specific binding to target DNA [129]. While these techniques offer high specificity, their cost and complexity can be limiting factors. Additionally, while the reversibility of affinity interactions aids in sensor regeneration, it may pose challenges in applications requiring ultra-stable immobilization. A comparative summary of these immobilization strategies, highlighting their key advantages and limitations in the context of biosensor performance, is provided in Table 5.

The development of engineered nanobody—single-domain antibody fragments has opened new possibilities for enhancing sensor sensitivity. Their smaller size, higher stability, and ease of functionalization make them ideal for high-density immobilization. Ma et al. (2023) reported a LOD of 0.01 ng/mL for SARS-CoV-2 spike protein using two nanobodies in tandem [130]. A comparative study on a paper-based immunosensor found that nanobody-based immobilization was 500 times more sensitive than IgG-based counterparts, achieving 0.035 μg/mL detection of aflatoxigenic fungi [131].

Scaffold-mediated orientation provides another path to enhanced sensor performance. Wen et al. (2023) employed nanofibrous membranes for the oriented immobilization of antibodies, increasing signal response by a factor of 10 and reducing the LOD to 0.48 pg/mL for parathion [132]. Rutten et al. (2020) applied 3D DNA origami scaffolds to elevate antibody orientation and spacing on microparticles, minimizing steric hindrance and optimizing analyte access—key to achieving high-fidelity sensing in multiplexed platforms [133]. Advances in nanoparticle coatings, nanorods, and viral nanoscaffolds have further facilitated the creation of high-density receptor attachment sites on sensing surfaces. Poghassian et al. showed that gold nanoparticles, when functionalized with ligands, can provide anchoring points for capture probes [134]. Their nanoscale dimensions enable precise spacing and orientation control, ensuring high receptor loading and sustained bioactivity. Koch et al. reported use of viral scaffolds like tobacco mosaic virus (TMV) for enzyme immobilization and their reliable repeated reuse in complex media [135].

Abouzar et al. reported that multilayer coatings of polyelectrolytes or dendrimers can enhance stability and probe functionality [126]. The structural flexibility of dendrimers, combined with the electrostatic binding properties of polyelectrolytes, ensures that receptors remain bioavailable and functional under the physiological conditions of bodily fluids [121]. Nam et al. (2024) utilized the electropolymerization of pyrrole (Ppy) to facilitate kinetically facile and high-density antibody immobilization on electrode surfaces. This one-minute process yielded a LOD of 3.13 pg/mL for α-fetoprotein, demonstrating both speed and efficacy [136]. Lin et al. (2018) employed boronic acid-containing linkers, enabling immobilization in seconds with densities exceeding 400 ng/cm^2^, a fourfold increase over traditional methods [137]. This approach is particularly attractive for scaling up rapid diagnostic platforms.

## 6. Future Directions

Capacitive sensors, ranging from interdigitated to vertically paired electrodes, have significantly advanced label-free detection since 1986. However, their application in biological fluids remains limited by unreliable sensor responses, non-selectivity, and poor longevity. Addressing the constraints imposed by Debye length in high-ionic-strength biofluids remains a persistent challenge. To improve the performance of capacitive sensors and mitigate the technical challenges of Debye length limitations, focus should be centered around (a) advancing nanostructured electrode configurations, such as nanopores and nanogaps, which can amplify the electric field strength, allowing biomarker-induced perturbations to overcome ionic screening; (b) developing novel polymer–electrode compositions, with tunable electrical conductivity, such that they can be integrated into electrode assemblies as mediators or extensions of the metallic electrode; (c) improving surface functionalization strategies, such that the structural integrity of capture probes is preserved and a compact, pinhole-free immobilization layer is fabricated; (d) adoption of signal-enrichment strategies like ACEK, which can significantly reduce the time required for testing and provide better provision for point of care diagnostics.

Building on these advancements, the next generation of capacitive biosensors will rely on the rational design of sensing electrodes, optimized surface morphology, seamless material integration, and robust surface immobilization.

## Figures and Tables

**Table 2:** Summary of specific and non-specific binding features upon intermolecular interactions and structural characteristics [56].

Features	Specific Binding	Nonspecific Binding
Intermolecular forces	Short-range forces (van der Waals/ acid base interactions)	Long-range forces (electrostatic/hydrophobic)
Structural (interface architecture)	Defined final interface and orientation	Interface/orientation not well-defined
	Evolved or designed binding sites	No evolved or designed binding sites
	Specific interactions are higher in affinity than all other binders to interface	Can still be selective (interactions of some proteins are favored)

**Table 3:** Criteria for the successful regeneration of a biosensor [56].

Attribute	Criteria
Signal loss between interrogation cycles	<5%
Number of continual cycles achieved	>10
Restoration of baseline signal	<±5%
Biosensor/transducer reconstruction	Avoided
Signal loss profile	Linear, allowing accurate calibration
Regeneration conditions	Explicitly listed with incubation time and full buffer components

**Table 4:** Immunosensors utilizing non-Faradaic EIS.

Biofluid	Electrode Design	Insulating Layer	Recognition Element	Disease	LoD	Ref., Year
Human Plasma	IDμEs	Polypyrrole	Antibody	Traumatic Brain Injury	0.184, 0.339 fg/mL	[98], 2025
Human Plasma	Glassy Carbon Electrode	rGO-AuNPs-Thionine	Antibody	Thrombotic Disorders	0.184, 0.339 fg/mL	[99], 2025
Synthetic Human Sweat	AuNP-functionalized LIG-IDE	MUA SAM Layer	Antibody	Cortisol	0.0085 nM	[100], 2025
Urine and Nasal Samples	IDEs	APTES	Aptamer	SARS-CoV-2	10 fg/mL	[101], 2025
PBS	Carbon SPEs	Acrylic Resin, Chitosan and Nitrocellulose Membrane	BSA Molecule	Anti-BSA Antibodies	2.5 mg/mL	[102], 2024
Human Serum	Au SPEs	Cys/DCC & DMAP	Yellow Fever Antigen	Yellow Fever Antibodies	96 ag/mL	[103], 2024
Saliva (spiked)	Au Working Electrode	SAM	Anti-A29 Monoclonal Antibody	Monkeypox (A29 Protein)	1.8 ng/mL	[104], 2024
Blood Serum	SPCEs with MNC substrate	SAM	Anti-CEA Antibodies	Cancer	9.04 pg/mL	[105], 2024
Artificial CSF	Carbon SPEs with Au Microflowers	SAM	Antibody	Parkinson’s	0.207 ng/mL	[106], 2024
Human Serum	Au IDEs	SAM; EDC/NHS	SARS-CoV2 Spike Protein	COVID-19	21 ng/mL	[107], 2024
Human Serum	Au SPEs	SAM	Aptamer	Zika Virus, Dengue	41.8 fg/mL	[108], 2024
Human Serum	rGO electrodes + AuNPs	Physiosorption	Antibody	Cancer	8.9 U/mL	[109], 2024
Serum	AuSPEs	Mesoporous Silica Nanoparticles (SiNPs)	Antibody	Ovarian Cancer	0.02–20 pM (HE4)	[110], 2023
Artificial Urine	IDEs	Ta_2_O_5_ + Biotin/ Streptavidin	Antibodies	Kidney Tubular Damage	0.5 ng/mL–8 ng/mL	[111], 2023
Serum	ITO electrode with APTES/nMoS_2_ NS@rGO Nanohybrid	APTES Silanization	Antibody	Cancer (Sp17 Biomarker)	0.13 ng/mL (Amperometric), 0.23 ng/mL (EIS)	[112], 2023
10% Human Serum	Au MEAs	SAM, EDC/NHS; SAM (MCH)	Nanobody; Aptamers	Interleukin-6, IL-6	10 pg/mL	[113], 2023
Human Serum	IMA	SAM (APTES, MUOH), EDC/NHS	(S-Protein) of SARS-CoV-2	SARS-CoV-2	0.4 BAU/mL	[114], 2023
Serum	Fe-Cu LDH/rGO Nanocomposite on PGE	Physiosorption	Anti-PSA Antibodies	Prostate Cancer	63.24fg/mL	[115], 2023
Artificial Human Saliva	Au Microelectrode Array	MCH Co-immobilization	Aptamer	NT-proBNP	$5.0\times 10^{-3}$–1.0 pg/mL; LOD = $5.0\times 10^{-3}$ pg/mL	[116], 2023
Viral Culture Fluid	IDEs + Parylene	PEDOT:PSS	Antibody	SARS-CoV-2 Nucleoprotein	4.1 ng/mL	[26], 2022
Serum	Au Nanostars	Prussian Blue Nanocubes, Graphene Oxide	Antibodies	Inflammation/ Septic shock	Linear Response with R^2^ > 0.99	[117], 2022
Serum	IDME	Carboxylayed CNFs + EDC/NHS	Taenia Solium Antigen (rT24H)	Anti-rT24H Antibodies	24.1 fg/mL	[55], 2022
Urine	Au SPEs	DSP Crosslinker	Antibody	IL6, IL8	1 pg/mL	[118], 2022
Human Plasma	IDEs	APTS-Pt/Ti–SiO_2_ IDCs	o-Aβ Aptamer, Antibody	Aβ42 Oligomers	0.1 fg/ mL	[119], 2022
Saliva	Au IDEs	SAM (L-Cys)	ACE2 protein	SARS-CoV-2	750 pg/μL/mm^2^	[120], 2022
Serum	ITO microdisk	β-CD/RGO nanohybrid	Antibody	Aβ40	0.69 fg/mL	[121], 2022
Blood	GrO-glazed DIDC	EDC-NHS	Antibody	SARS-CoV-2	1.0 fg/mL	[122], 2021
Diluted Serum	Au IDEs	SAM	ssDNA	miRNA-16b	1.0 fg/mL	[51], 2021
DI Water	Pt/Ti IDEs	APTES	DNA Probe	SARS-CoV-2	10 ng/mL	[123], 2021
PBS	Au electrodes	SAM (Fc-Glu-(Ala)n-Cys-NH_2_)	Antibody	Inflammation	240 pg/mL	[124], 2021

**Table 5:** Comparison of immobilization strategies for bioreceptors in capacitive biosensors.

Immobilization Strategy	Advantages	Drawbacks
Covalent Binding	Strong and stable immobilization; low risk of receptor loss; avoids physical desorption	May reduce receptor conformational flexibility; steric hindrance potential
Physical Adsorption	Simple, low cost, and easily reversible	Weak attachment; high risk of desorption; unstable under high ionic strength conditions
Polyelectrolyte Multilayers	Enhances receptor density; maintains receptor hydration; improves stability and bioactivity	May lead to receptor aggregation; variability in reproducibility; complex fabrication processes
Self-Assembled Monolayers	Precise surface control; reduces nonspecific adsorption; improves receptor orientation	Requires optimized surface chemistries; fabrication can be time-intensive
Nanoparticle Assisted Immobilization	High receptor density; enhanced stability and signal amplification; potential for reusable surfaces	Additional functionalization required for nanomaterials; scalability challenges
Encapsulation Strategies (Zwitterionic Polymers)	Protects bioreceptors from harsh environmental factors; ensures hydration; extends receptor longevity	Potential diffusion limitations may reduce reaction kinetics; integration with sensors can be complex
Affinity Based (Protein A/G-Based Binding)	Exploits highly specific affinity interactions; ensures correct Fab orientation of antibodies	Primary reliance on affinity stability; not suitable for all bioreceptors

## Abbreviations

The following abbreviations are used in this manuscript:

EIS	Electrochemical Impedance Spectroscopy
f-EIS	Faradaic-Electrochemical Impedance Spectroscopy
nf-EIS	Non Faradaic-Electrochemical Impedance Spectroscopy
LoD	Limit of Detection
EDL	Electrical Double Layer
IDEs	Interdigitated Electrodes
VPsE	Vertically Paired Electrodes
MAIDEs	Microneedle Array Integrated with IDEs
SNR	Signal-to-Noise Ratio
ACEK	Alternating Current Electrokinetic
IDMEs	Interdigitated Microelectrodes
DEP	Dielectrophoretic
SAM	Self-Assembled Monolayer
MIPs	Molecularly Imprinted Polymers
ISF	Insterstitial Fluid
BSA	Bovine Serum Albumin
